# Comparative emission and energy performance of biochar production versus in-situ burning of Gorean agrobyproducts

**DOI:** 10.1371/journal.pone.0346041

**Published:** 2026-04-24

**Authors:** Sunyong Park, Kwang Cheol Oh, Seok Jun Kim, Padam Prasad Paudel, Seon Yeop Kim, Kyeong Sik Kang, Sunhwa Ryu, DaeHyun Kim

**Affiliations:** 1 Forest Industrial Materials Division, National Institute of Forest Science, Dongaemungu, Seoul, Republic of Korea; 2 Korea Research Institute on Climate Change, Subyeongongwon-gil, Chuncheon-si, Republic of Korea; 3 Department of Environmental Research, Korea Institute of Civil Engineering and Building Technology, Goyang, Korea; 4 Department of Interdisciplinary Program in Smart Agriculture, Kangwon National University, ChunCheon, Korea; Universiti Teknologi Petronas: Universiti Teknologi PETRONAS, MALAYSIA

## Abstract

Uncontrolled open-field burning of agricultural residues remains a pervasive source of air pollutants and greenhouse gases in Korea, particularly for high-burning-ratio crops such as perilla, pepper, pear, grape, and apple residues. This study provides a comprehensive assessment of the environmental and energy implications of replacing open-field burning with biochar production from these major residue streams. First, mass- and energy-normalized emission factors for CO, CH₄, CO₂, NOₓ, N₂O, and particulate matter were quantified for both in-situ combustion and biochar combustion scenarios. Although biochar exhibited higher mass-based emission factors due to carbon and nitrogen enrichment during pyrolysis, the substantially lower mass required to deliver equivalent energy output resulted in markedly reduced annual emissions. System-level calculations showed that shifting from open-field burning to biochar conversion decreased CO, CH₄, and CO₂ emissions by 40.6–46.5%, NOₓ and N₂O emissions by 29.9–92.7%, and particulate emissions by 14.7–86.6%. Energy analysis demonstrated that biochar production is energetically favorable, with energy inputs of 2.65–3.50 MJ/kg and energy return on investment (EROI) values ranging from 8.57 to 10.06, exceeding the conventional viability threshold of 3.0. Apple pruning residues showed the highest biochar and net energy potentials, corresponding to 36.45 GWh/yr of electricity generation when co-fired in existing thermal power plants. Finally, greenhouse gas impacts were evaluated using 100-year global warming potential metrics. The biochar pathway reduced annual climate impacts by approximately 192,967 tCO₂eq/yr relative to baseline burning, translating to an estimated carbon-credit benefit of 4.44 billion KRW under 2020 market conditions. Overall, the findings indicate that biochar conversion provides a viable and impactful strategy for mitigating emissions and enhancing renewable energy recovery from agricultural residues in Korea.

## Introduction

Energy consumption has increased in tandem with the expansion of industry. The utilisation of a significant amount of fossil fuels was a consequence of this energy consumption trend. This resulted in significant environmental contamination. At present, the United Nations employs the term “global boiling” rather than “global warming” [1]. To address this issue, the Paris Agreement was signed in 2015 to prevent the Earth’s average temperature rising [2,3]. Korea’s 2030 business-as-usual (BAU) reduction target of 37% has been revised to a specific absolute value, aiming for a reduction of 24.4% compared to the emissions level in 2017 [4,5]. Korea must urgently reduce GHG emissions under the 2030/2050 targets, but agricultural residue burning remains a persistent overlooked emission source.

In agricultural production, pollutants are released into the atmosphere through several pathways, including the use of agricultural machinery and transportation [6–8]. Of them, the act of illegally burning waste in rice paddies or fields is being identified as a significant issue [6,9,10]. After the conclusion of the current harvest season, a variety of byproducts remain in rice paddies or fields. If straw or rice husks have a purpose like animal feed or livestock bedding, they are gathered and used. However, agricultural byproducts that do not have a specific demand are left in their original location and burned in rice paddies or fields before the busy farming season begins [11–15]. Unlike power plants, this particular kind of incineration lacks the advantage of post-processing methods like desulfurization or denitrification, resulting in the dispersion of all produced pollutants into the atmosphere. Furthermore, it is identified as a contributing factor to extensive flames, such as forest fires, when sparks are carried by the dry weather and early spring winds to other locations [16]. Wildfire data indicate that 58% of a forest fire recorded from 2012 to 2021 happen during the spring season [17]. Additionally, an average of 512 incidents of agricultural waste burning were reported throughout this period. This accounts for about 20% of the forest fire that occurred during the period.

Several research efforts have been carried out to mitigate this supplementary environmental contamination and make use of remaining agricultural residue. Ju et al. (2024) analysed chemical characteristics of algrobyproduct and evaluated fuel property [18]. The agrobyproducts were met Korea solid refuse fuel (SRF) standards but the biomass only satisfied Level 3 of the European standard for SRF due to its low calorific value. Jagodzińska et al (2020) investigated the potential uses of torrefied agricultural residues, finding that biochar could be used in agriculture, while the condensable volatiles were highly toxic and may serve as pesticides or anaerobic digestion substrates after detoxification [19]. Park et al. (2023) converted agrobyproduct into biochar and analysed for agricultural fertiliser or solid biofuel [20]. Kim et al. (2021) conducted surface torrefaction on agricultural byproduct which could be addressing conventional torrefaction [21]. Anand et al. (2022) reported possibility of reducing air pollutant by converting biochar in northern India [22]. Numerous studies have sought to use agricultural byproducts. But there is currently less study that provides an integrated comparison of emissions, greenhouse gas impacts, and energy potential between open-field burning and biochar conversion of agricultural residues.

Biochar production offers a realistic and technologically compatible alternative to open-field burning. The pyrolysis process stabilizes biomass carbon into aromatic structures, substantially reducing the fraction of carbon released as CO2, CO, and CH4 [23]. As volatile and nitrogen-containing components are removed, biochar combustion emits fewer air pollutants per unit energy than raw biomass [24]. Furthermore, biochar exhibits fuel properties comparable to low-rank coal, enabling direct use in existing boiler–steam turbine systems without major retrofitting [25]. Because agricultural residues in Korea already undergo natural drying and are logistically centralized during harvest, biochar conversion can be integrated efficiently into existing residue collection systems [26,27]. Consequently, biochar provides simultaneous benefits in GHG mitigation, air-pollutant reduction, renewable energy generation, and carbon-credit acquisition, making it a uniquely viable pathway compared with other residue-utilization options [28–32].

So, this study specifically chose biomass that is commonly burned in open fields, based on prior research. It then verified the decrease in air pollutants when this biomass is turned into biochar, and examined the advantages that might be gained by connecting this process to carbon emissions.

## Materials and methods

### System boundary and analysis outline

This study adopts an energy-based system boundary that includes only the thermal conversion and combustion stages of agricultural residues. The analysis compares the current baseline practice of open-field burning with an alternative pathway in which residues are converted into biochar through slow pyrolysis and the resulting biochar is subsequently combusted to generate electricity.

In the baseline pathway, residues are assumed to be burned at the field edge without collection, transport, or emission-control measures. The system boundary therefore consists solely of natural drying prior to burning and uncontrolled on-site combustion. Emissions of CO, CO_2_, CH_4_, NO_X_, N_2_O, and particulate matter are quantified using an elemental balance approach consistent with IPCC guidelines. In the biochar pathway, residues are assumed to be available at the conversion site, and the boundary encompasses only thermochemical conversion and subsequent combustion. Slow pyrolysis is used to produce biochar, which is then combusted in a boiler–steam turbine system to generate electricity. Co-products such as bio-oil and syngas are excluded because the analysis focuses exclusively on the biochar-to-electricity route. Facility construction and decommissioning are also outside the scope.

This boundary definition enables a direct, energy-equivalent comparison of combustion-stage emission intensity between uncontrolled burning and thermochemically controlled conversion, without the confounding influence of highly site-specific upstream logistics.

### Agricultural byproduct potential

Crop leftovers refer to the residual remains that are left behind after the process of crop cultivation. The gross residue potential of a crop may be assessed by considering three factors: the area of land used for cultivating the crop (A), the amount of crop produced per year (P), and residue production conversion factor (RPCF) [22,33]. Based on the parameters given, the estimation of gross crop residue per year (CR) production may be calculated as Eq (1).


CR=A×P×RPCF (1)

where, A is the area of land used for cultivating the crop (ha), P refers to the amount of crop produced per year [ton/ha ∙ yr], RPCF stands for residue production ratio [-] and CR is the estimation of gross crop residue [ton/yr].

### Emission due to in–situ burning

The total quantity of agricultural byproduct resulting from in-situ burning was calculated using Eq. (2).


$$CR_{B}=CR\times DM\times \eta_{\text{B}}\times X_{Burnt}$$ (2)

where 𝐶𝑅_𝐵_ is the total mass of biomass burned in situ (ton yr ⁻ ¹), 𝐷𝑀 is the dry matter fraction of the residue (–), *η*_*B*_ is the combustion efficiency (–), and 𝑋__𝑏_𝑢𝑟𝑛𝑡_ is the fraction of crop residues burned in situ (–).

In this study, dry matter was calculated by subtracting ash content from the total mass, assuming that ash represents the non-combustible fraction of the residue. The combustion efficiency (η_B)_ represents the fraction of dry combustible matter effectively oxidized under open-field burning conditions. Although combustion efficiency can vary depending on fuel properties and environmental factors, reported values in the literature generally fall within a relatively narrow range of 0.88–0.95. To ensure methodological consistency and comparability across biomass types, a representative value of 0.92 was uniformly applied in this study. In the case of X_burnt_, the ratio of each biomass was shown according to previous research.

Emission factors for agricultural biomass combustion were calculated using the IPCC-aligned elemental balance method, which is widely applied for cases where direct emission measurements are unavailable. This approach allows consistent estimation of CO, CO₂, CH₄, NOₓ, and N₂O based on biomass elemental composition [34]. These pollutants were calculated using Eqs. (3–10) in Table 1 [35,36].

**Table 1:** Equation summary of emission factor.

Emission Factor	Equation	
EF_C_ [kg kg^-1^]	${EF}_{C}=\text{0}.\text{8}\text{8}\times C$	(3)
EF_CO_ [kg kg^-1^]	$EF_{CO}=\left(\frac{\text{2}\text{8}}{\text{1}\text{2}}\right)\times {EF}_{C}\times (C_{CO}/C)$	(4)
EF_CH4_ [kg kg^-1^]	$EF_{CH\text{4}}=\frac{\text{1}\text{6}}{\text{1}\text{2}}\times {EF}_{C}\times (C_{CH\text{4}}/C)$	(5)
EF_CO2_ [kg kg^-1^]	$EF_{CO\text{2}}=\frac{\text{4}\text{4}}{\text{1}\text{2}}\times \left({EF}_{C}-\frac{\text{1}\text{2}}{\text{2}\text{8}}\times CO-\frac{\text{1}\text{2}}{\text{1}\text{6}}{EF}_{CH\text{4}}-\frac{\text{2}\text{6}.\text{4}}{\text{3}\text{1}.\text{4}}\times {EF}_{NMVOC}\right)$	(6)
EF_NOX_ [kg kg^-1^]	$EF_{NOX}=\frac{\text{4}\text{6}}{\text{1}\text{4}}\times {EF}_{C}\times \frac{N}{C}\times (N_{NOx}/N)$	(7)
EF_N2O_ [kg kg^-1^]	$EF_{N\text{2}O}=\frac{\text{4}\text{4}}{\text{2}\text{8}}\times {EF}_{C}\times \frac{N}{C}\times (N_{N\text{2}O}/N)$	(8)
EF_dust_ [kg kg^-1^]	$EF_{dust}=\text{1}.\text{5}\times Ash\times \frac{\text{1}\text{0}\text{0}-\eta_{o}}{\text{1}\text{0}\text{0}-k}$	(9)
E_CV_ [kg MJ^-1^]	$E_{CV_{i}}=\frac{EF_{i}}{CV}$	(10)

EF refers to emission factors of subscription [kg/kg], Ash stands for ash content [%], η_o_ is dust removal efficiency (for biomass 20%) and k refers to content of flammable parts in the dust (for biomass 5%). CV refers calorific value of agrobyproduct. For subscription, C, CO, CH_4_, CO_2_, NO_X_, N_2_O, NMVOC and dust stand for carbon, carbon monoxide, methane, carbon dioxide, nitrogen oxides, nitrogen dioxide and non-methane volatile organic compounds, respectively. Subscription i represents the pollutants (i.e., CO_2_, CO, CH_4_).

Several greenhouse gases and pollution are released straight into the air when agrobyproducts were burned on-site. Eq. (3) was used to measure the amount of pollution (E_m_) released when the biomass were burned on-site. Also, to compare the amount of pollutants per unit calorific value (E_cv_), Eq. (11) was used.


$$E_{m}=CR_{B}\times EF$$ (11)

The total carbon dioxide equivalent of in-situ combustion of agrobyproduct was calculated using the calculated pollutant emissions and global warming potential as Eq. (12)


$$GWP_{T}=\sum \left(EF_{i}\times GWP_{i}\right)$$ (12)

where, GWP_T_ refers global warming potential of residue burning in CO_2_ equivalence (T CO_2eq_), EF and GWP are respective emission and GWP of pollutions. Subscription i represents the pollutants (i.e., CO_2_, CO, CH_4_). The GWP of CO, CH_4_, CO_2_, NO_X_, and N_2_O are known to be 1.9, 21, 1, 10, and 310, respectively [37–40].

To improve interpretability and enable basis comparison across biomass types, emissions were additionally normalised per unit dry residue mass (kg_pollutant_ ton^-1^) and per unit useful energy output (kg_pollutant_ GJ^-1^). This approach allows separation of scale effects driven by residue availability from intrinsic emission intensity determined by fuel composition.

### Energy analysis and electricity generation potential of biochar

For biochar production, ambient and process temperature were set as 25℃ and 500℃, respectively. The energy required for biochar synthesis at temperature ‘T’ via pyrolysis was taken as input energy and computed using the following Eq. (13).


$$E_{in}=\eta_{h}\times (m_{wet}\times \left(H_{T}-H_{amb}+H_{vap}\right)+m_{dry}\times C_{p}\times \left(T-T_{amb}\right))$$ (13)

where, m_wet_ and m_dry_ refer to mass for moisture and bone-dried biomass [kg], H_T_ and H_amb_ stand for specific enthalpy of water at the process and ambient temperature (3488 and 104.92 kJ/kg), H_vap_ means latent heat of water vaporization (2260 kJ/kg). C_P_ refers specific heat capacity of the biomass. η_h_ stands for heat transfer efficiency, which was set as 0.6 (60%). The biomass exposed to the field undergoes natural drying, resulting in a reduction of its moisture content to below 20%. To obtain the most conservative estimate, the moisture content was assumed to be 20%. In general, when the temperature of biomass rises, devolatilization takes place, leading to a decrease in C_P_. For this investigation, it turned out to represent it as a constant.

The Energy Return on Investment (EROI) is an important indicator for evaluating the sustainability of biochar as a fuel [41]. EROI is a measure of the ratio between the energy value of a fuel and the energy expended in obtaining and distributing it. EROI investigation was conducted to ascertain the energy utilisation advantages of biochar following the process of pyrolysis. EROI has been calculated using Eq. (14).


$$EROI=\frac{CV_{T}}{E_{in}}$$ (14)

where, EROI stands for energy return on investment [-], CV_T_ refers to calorific value of biochar [MJ/kg].

Total biochar potential (BP_T_) of crop residues was estimated by following Eq. (15):


$$BP_{T}=MY_{T}\times CR_{B}$$ (15)

where, BP_T_ stands for biochar potential [ton/yr], MY_T_ refers to mass yield of biochar [%].

The suggested agricultural residue management approach includes power generation from biochar created from byproducts. It was determined that the energy needed for biochar conversion was sourced from power produced in the cycle before. As a result, The electricity generation potential (EGP) has been determined using net energy potential (NEP), which is calculated by balancing input energy in biochar as Eq. (16).


$$NEP_{T}=BP_{T}\times CV_{T}-E_{in}\times CR_{B}$$ (16)

where, NEP_T_ refers to net energy potential [TJ/yr]

The Boiler-steam turbine system is the optimal technology for generating power from biochar. It has similarity to coal-based thermal power plants, and has attained an electricity generating efficiency of 30–44%. The calculations in this study have been conducted using the Boiler-steam turbine system, which has been proven to be the most reliable, widely used, and cost-effective solution for this purpose [42,43]. EGP of biochar was determined by using Eq. (17).


$$EGP=\frac{\eta \times NEP_{T}}{\text{1}\text{0}\text{0}}$$ (17)

where, EGP stands for electricity generation potential [TJ/yr], η refers to electricity generating efficiency in Korea. The power plant efficiency, denoted by η, has a global variation of 30–50%. The projected thermal power plant efficiency in Korea is 41% [44]. The current research has taken into account a power plant efficiency of 41%.

### Emission due to biochar combustion

It was assumed that pollutants generated during the biochar process were purified within the process. Therefore, only the pollutants generated during biochar combustion were considered and expressed in Eq (18).


$$E_{Bm}=BP_{T}\times EF$$ (18)

BP_T_ refers biochar potential [ton/yr], EF stands for the emission factors of the different pollutants that were released.

The suggested pathway (SP)—biochar combustion for power generation—has had its GWP compared to the standard operating procedure, which is in-situ burning. Additionally, the suggested scenario’s carbon footprint and the GWP for in-situ agrobyproduct were taken into account. Carbon credit, calculated using the following Eq (19), represents the emission reduction resulting from the suggested approach.


$$Carbon\;credit=GWP_{BAU}-GWP_{SP}$$

where, GWP_BAU_ and GWP_SP_ stand for the GWP due to in-situ burning of agrobyproduct on business as usual and biochar combustion, respectively.

## Results & discussion

### Target biomass selection and biomass potential

In Korea, approximately 13.6% of agricultural residues are burned in situ [45], with substantially higher burning ratios reported for specialty crops, seasoning crops, and fruit tree residues (21.0%, 20.2%, and 14.4%, respectively). Based on their elevated in-situ burning rates and their documented contribution to rural air-quality deterioration, perilla (PR), pepper (PP), pear (PE), grape (GP), and apple (AP) residues were selected as the target biomass streams in this study. These residues not only represent environmentally significant open burning practices but also account for a substantial portion of national agricultural byproduct generation. In addition, their lignocellulosic composition and manageable moisture characteristics make them suitable candidates for thermochemical conversion. Collectively, they provide a representative basis for evaluating both environmental burden and technical feasibility in biochar-based mitigation strategies. Table 2 was summarised crop cultivation area, crop production per area, conversion factor and byproduct production per year in 2020. Among the selected residues, PR and PE had the largest cultivation areas, whereas PP had the smallest. In contrast, PP, GP, and AP exhibited much higher crop production per unit area (15.54–18.09 ton/ha·yr) than PR and PE (1.07–1.93 ton/ha·yr). Consequently, annual residue production (CR) was highest for AP, followed by GP and PR, while PP yielded the lowest CR due to its limited cultivation area. Taken together, these five residue types represent a nationally significant resource stream, combining high burning propensity with substantial generation volumes. They are also among the most frequently cited sources of open-field burning complaints in rural areas, highlighting their priority in air-pollution mitigation efforts. In addition, their lignocellulosic characteristics and naturally low moisture contents make them suitable feedstocks for thermochemical conversion. For these reasons, the selected residues constitute an appropriate focus for evaluating the energy and environmental implications of replacing open-field burning with biochar production.

**Table 2:** Basic agricultural data of selected biomass in 2020 [27,46,47].

	A [ha]	P [ton/ha ∙ yr]	RPR [–]	CR [ton/yr]
PE	31,146	1.93	2.60	156,290.63
PR	36,111	1.07	6.14	237,242.05
PP	8,530	15.54	0.66	86,956.87
GP	9,988	16.61	1.56	259,136.86
AP	23,330	18.09	1.32	555,404.25

### Emission of greenhouse gases and pollutants

Dry matter (DM), burning efficiency (η_B_), and the in-situ burning fraction (X_burnt_) were obtained from previous field studies [45,46,48–50] and used to quantify the total mass of residues burned in the field (CR_B_) using Eq. (2). Collectively, these parameters determine how much of the generated residue becomes an effective combustible mass contributing to open-field emissions. Calculated total mass of CR_B_, DM, η_B_ and X_burnt_ were summarised as Table 3. Although PP exhibited the highest DM due to its low ash content, AP and PE ultimately contributed the CR_B_ because of their substantial residue generation and relatively high X_burnt_ values. In contrast, GP showed a relatively low in-situ burning fraction (7.8%), resulting in a smaller contribution to field emissions despite its considerable residue generation. These differences indicate that both residue quantity and burning propensity jointly determine the magnitude of field emissions.

**Table 3:** Summary data for calculating total mass of in-situ biomass [45,46,48–50].

	PE	PR	PP	GP	AP
DM [–]	0.9271	0.9186	0.9660	0.9417	0.9610
η_B_ [–]	0.9200				
X_burnt_ [–]	0.3360	0.2000	0.2750	0.0780	0.1820
CR_B_ [ton/yr]	44,790.57	40,099.22	21,252.08	17511.53	89,370.01

Table 4 presents the predicted pollutant emissions during in-situ biomass combustion calculated using Eqs. (3–10), expressed on both a mass-based (kg/Mg residue) and an energy-normalised basis (kg/GJ). On a mass basis, CO and CO_2_ accounted for the dominant share of gaseous emissions across all residues. PP exhibited the highest CO (61.48 kg/Mg) and CO_2_ (1505.42 kg/Mg) emissions, reflecting its elevated carbon content. In contrast, PR and PE showed lower carbon-based emissions but differed in particulate formation. Dust emissions were highest for PR (10.28 kg/Mg), consistent with its higher ash fraction, while PP produced the lowest dust emission (4.29 kg/Mg). Nitrogen-related emissions (NO_X_ and N_2_O) were most pronounced for PE (6.56 kg/Mg NO_X_; 0.18 kg/Mg N_2_O), corresponding to its relatively higher nitrogen content.

**Table 4:** Release of pollutants during the in-situ biomass burning per unit mass and unit calorific value.

	PE		PR		PP		GP		AP	
	kg/Mg	kg/GJ	kg/Mg	kg/GJ	kg/Mg	kg/GJ	kg/Mg	kg/GJ	kg/Mg	kg/GJ
CO	53.32	2.90	53.09	2.90	61.48	2.98	54.71	2.91	55.26	2.92
CH_4_	2.54	0.14	2.53	0.14	2.93	0.14	2.61	0.14	2.63	0.14
CO_2_	1305.70	71.01	1299.97	70.95	1505.42	72.87	1339.79	71.36	1353.07	71.49
NO_X_	6.56	0.36	2.29	0.13	2.58	0.12	3.42	0.18	3.95	0.21
N_2_O	0.18	0.01	0.06	0.00	0.07	0.00	0.09	0.01	0.11	0.01
Dust	9.21	0.50	10.28	0.56	4.29	0.21	7.36	0.39	4.93	0.26

When emissions were normalised by energy content, differences among residues became substantially smaller. For instance, CO_2_ emissions ranged narrowly between 70.95 and 72.87 kg/GJ, and CO varied only from 2.90 to 2.98 kg/GJ across all residue types. This convergence indicates that PP’s higher heating value compensates for its higher mass-based emissions, resulting in comparable energy-based emission intensities. Similarly, CH_4_ emissions remained nearly identical at approximately 0.14 kg/GJ for all residues.

These results demonstrate that while mass-based emission factors are strongly influenced by elemental composition—particularly carbon, nitrogen, and ash content—energy-normalised comparisons reduce apparent inter-residue variability. The dual-basis evaluation therefore distinguishes scale and compositional effects from intrinsic emission intensity per unit useful energy.

**Fig 1:**
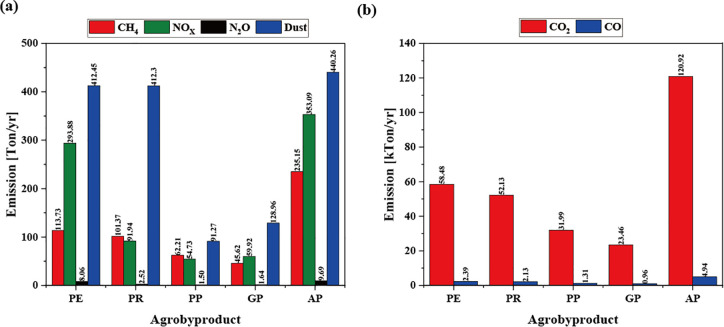
Pollutants emission per year (a) CH_4_, NOX, N_2_O, Dust (b) CO_2_, CO.

The total annual emissions were predicted using the amount of biomass incinerated in the field and the pollutant emissions by biomass. Fig 1 presents the calculated annual pollutant emissions for each agrobyproduct based on Eq. (11). Among the residues, AP exhibited the highest total emissions. This reflects not only relatively high emission intensity per unit mass but also its substantial residue production and high in-situ burning fraction. Across all residues, CO_2_ was the dominant pollutant, ranging from 23.46 to 120.92 kT yr^-1^, followed by CO at 0.96–4.94 kT yr^-1^. Emissions of NO_X_ and N_2_O ranged from 54.73 to 353.09 ton yr^-1^ and 1.50 to 9.69 ton yr^-1^, respectively. Dust emissions were comparable for PE, PR, and AP, within 412.30–440.26 ton yr ⁻ ¹. Notably, PE and PR exhibited relatively high particulate emissions despite lower total residue production compared with AP, primarily due to their higher ash content.

Overall, AP and PE contributed most significantly to total field emissions owing to their large residue volumes and elevated in-situ burning ratios. PP, in contrast, produced a smaller total burned mass but generated comparatively high CO and CO_2_ emissions per unit mass due to its higher carbon content. PR showed a disproportionate contribution to particulate emissions, consistent with its higher ash fraction.

The variation among residues is therefore driven primarily by elemental composition and combustion characteristics rather than calorific value alone. Carbon-rich residues tend to yield higher CO and CO_2_ emissions, nitrogen-containing components influence NO_X_ and N_2_O formation, and elevated ash fractions are associated with increased particulate matter generation. These results underscore that emission intensity differences are composition-driven and support the need for residue-specific mitigation strategies.

### Energy analysis of Biochar production

The total energy input for biochar production (E_in_) was computed as the sum of the sensible heat required to increase the temperature of dry biomass (E_B_, determined by the specific heat capacity C_P_) and the latent and sensible heat associated with moisture removal (E_m_), as defined in Eqs. (13–17) and summarised as Table 5 [51,52]. Across the five residues, the total energy input ranged from 2.65 to 3.50 MJ/kg, driven primarily by differences in C_P_ and moisture-related energy demand, rather than by variations in heating value. The energy return on investment (EROI) ranged from 8.57 to 10.06, substantially exceeding the commonly cited minimum threshold of 3.0 for energy systems, indicating that biochar production from these residues is energetically favourable. Based on the mass yield of the previous study, the biochar potential (BP_T_) was predicted. The biomass with the highest potential was AP. This was determined as it produces the greatest byproducts. Regarding PE and PR, the potentials were close to equal at 12,678.75 and 11,885.41 tons/yr respectively. This was due to high field incineration rate of PE. PP and GP showed relatively low potentials at 4,824.22 and 5,428.58 tons/yr, respectively. In BC_EP_, AP showed the highest potential with 632.90 TJ/yr. However, PP showed the lowest BC_EP_, showing 138.63 TJ/yr. For NEP_T_, the difference between AP and other biomasses decreased. Nevertheless, it was found to have the highest potential among these biomasses, showing 320.01 TJ/yr. When this biochar was used in existing thermal power plants, the electricity production was 8.45–36.45 GWh/yr.

**Table 5:** Summary data for energy analysis.

	PE	PR	PP	GP	AP
CV_Raw_ [MJ/kg]	18.39	18.32	20.66	18.77	18.93
E_m_ [kJ/kg]	1128.62				
C_P_ [kJ/kg∙K]	1.2660	1.2200	1.8143	1.7980	2.5580
E_B_ [kJ/kg]	481.08	463.60	689.45	683.24	972.04
E_in_ [MJ/kg]	2.68	2.65	3.03	3.02	3.50
CV_Char_ [MJ/kg]	26.84	26.70	28.74	27.34	30.01
EROI [–]	10.00	10.06	9.48	9.05	8.57
MY [%]	28.53	29.64	22.70	31.00	23.60
BP_T_ [ton/yr]	12,778.75	11,885.41	4,824.22	5,428.58	21,091.32
BC_EP_ [TJ/yr]	342.92	317.40	138.63	148.40	632.91
NEP_T_ [TJ/yr]	222.75	210.99	74.23	95.51	320.01
EGP [GWh/yr]	25.37	24.03	8.45	10.88	36.45

### Emission of biochar combustion

Based on these energy results, the following section evaluates how replacing open-field burning with biochar production and combustion affects pollutant emissions at the system level. Biochar combustion yielded higher mass-based emission factors for all pollutants due to the concentration of carbon and nitrogen during pyrolysis. However, because substantially less mass is required to produce the same energy output, annual emissions under the biochar pathway were markedly lower than those from open-field burning. The results were summarised as Table 6. During pyrolysis, devolatilization removes oxygenated volatiles and enriches the remaining solid in fixed carbon and nitrogen-containing structures. This concentration effect increases the heating value of the char and elevates mass-based emission factors of CO₂, CO, NOₓ, and N₂O. Despite this increase, the reduced mass requirement per GJ leads to lower total annual emissions at the system level. The annual emissions were calculated by considering these emissions together with the biochar potential. The annual emissions of CO, CH_4_, CO_2_, NO_X_, N_2_O, and dust were calculated using Eq. (18). The results were summarised as Table 7. Despite the higher mass-based emission factors, annual emissions decreased substantially when replacing open-field burning with biochar conversion: CO, CH_4_, and CO_2_ decreased by 40.6–46.5%, NO_X_ and N_2_O by 29.9–92.7%, and dust by 14.7–86.6%. Overall, these results demonstrate that biochar conversion substantially reduces system-level pollutant burdens, even though biochar combustion exhibits higher mass-based emission factors than raw biomass.

**Table 6:** Pollutant emission calculation during biochar combustion in per unit mass and unit calorific value using Eq. (3–10).

	PE		PR		PP		GP		AP	
	kg/Mg	kg/GJ	kg/Mg	kg/GJ	kg/Mg	kg/GJ	kg/Mg	kg/GJ	kg/Mg	kg/GJ
CO	83.66	3.12	83.20	3.12	90.49	3.15	85.46	3.13	95.06	3.17
CH_4_	3.98	0.15	3.96	0.15	4.31	0.15	4.07	0.15	4.53	0.15
CO_2_	2,048.77	76.35	2037.31	76.29	2,215.91	77.11	2,092.82	76.56	2,327.84	77.57
NO_X_	6.88	0.26	3.39	0.13	10.51	0.37	8.22	0.30	9.56	0.32
N_2_O	0.19	0.01	0.09	0.00	0.29	0.01	0.23	0.01	0.26	0.01
Dust	4.75	0.18	30.03	1.12	13.78	0.48	9.98	0.37	10.89	0.36

**Table 7:** Annual emission during biochar combustion using Eq. (18).

	PE	PR	PP	GP	AP
CO	1,069.14	988.83	436.55	463.95	2,004.96
CH_4_	50.91	47.09	20.79	22.09	95.47
CO_2_	26,180.74	24,214.23	10,690.04	11,361.03	49,097.18
NO_X_	87.90	40.25	50.71	44.62	201.63
N_2_O	2.41	1.10	1.39	1.22	5.53
Dust	60.69	356.86	66.48	54.17	229.65

### GWP changes according to pyrolysis

**Fig 2:**
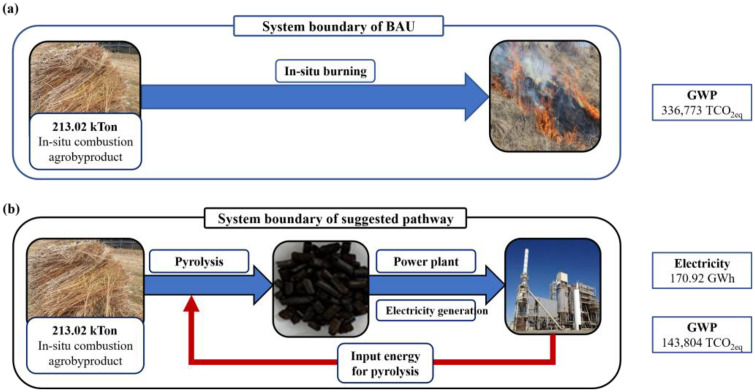
The system boundary of (a) BAU and (b) suggested pathway.

**Fig 3:**
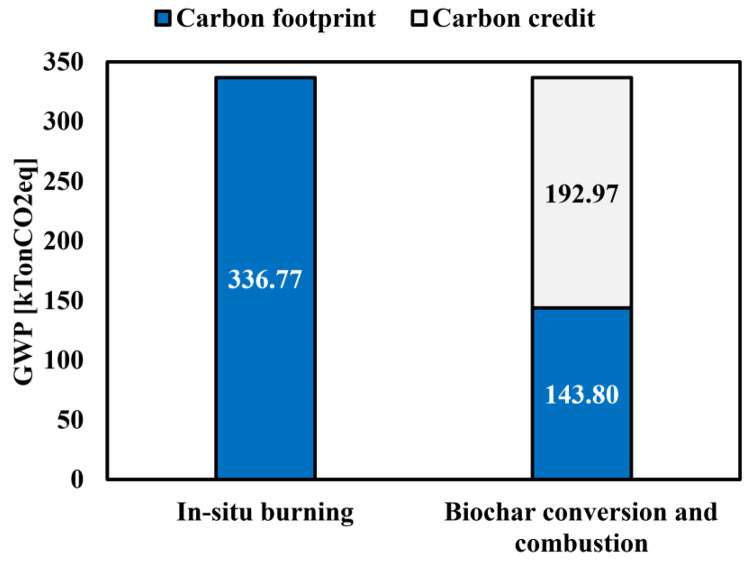
Carbon footprint and carbon credit of in-situ burning (BAU) and biochar conversion and combustion (suggested pathway).

The global warming potential (GWP) was evaluated in terms of 100-year CO_2_-equivalent (CO_2eq_) using Eq. (16). Annual emissions of CH_4_ and N_2_O from each pathway were multiplied by their respective GWP factors and added to direct CO_2_ emissions to derive the total CO_2eq_ for each residue. The CO_2eq_ was calculated annual emission pollutant and each pollutant GWP. Results of CO_2eq_ were summarized in Table 8. As shown in Table 8, AP exhibited the highest CO_2eq_ under the BAU scenario (baseline open-field burning) due to its large residue generation and substantial in-situ burning fraction. PE and PR followed, reflecting their intermediate levels of residue production and burning propensity, whereas PP and GP contributed comparatively smaller shares. System boundary for GWP estimate of business as usual (BAU) and suggested pathway was depicted in Fig 2(a),(b), respectively. The total GWP of the baseline open-field burning (BAU) system was estimated at 336,771 tCO_2eq_/yr (Fig 3). When the same residues were instead converted into biochar and combusted (BCC pathway), the total GWP decreased to 143,804 tCO_2eq_/yr. This corresponds to an absolute reduction of 192,967 tCO_2eq_/yr, equivalent to a 57.3% decrease relative to BAU. Applying the 2020 Korean Allowance Unit (KAU20) price of 23,000 KRW per tCO_2eq_/yr, the avoided emissions of 192,967 tCO_2eq_/yr translate into an annual carbon-credit revenue of approximately 4.44 billion KRW.

**Table 8:** GWP result for raw biomass and biochar combustion.

	PE		PR		PP		GP		AP	
	RAW	Biochar	RAW	Biochar	RAW	Biochar	RAW	Biochar	RAW	Biochar
GWP_CO_ [TCO_2eq_/yr]	4,537	2,031	4,044	1,878	2,482	829	1,820	881	9,382	3,809
GWP_CH4_ [TCO_2eq_/yr]	2,388	1,069	2,128	988	1,306	436	958	463	4,938	2,004
GWP_CO2_ [TCO_2eq_/yr]	58,483	26,180	52,127	24,214	31,993	10,690	23,461	11,361	120,923	49,097
GWP_NOX_ [TCO_2eq_/yr]	2,938	879	919	402	547	507	599	446	3,530	2,016
GWP_N2O_ [TCO_2eq_/yr]	2,499	747	782	342	465	431	509	379	3,003	1,715
Total GWP [TCO_2eq_/yr]	70,845	30,906	60,000	27,824	36,793	12,893	27,347	13,530	141,776	58,641

It should be noted that the present GWP and carbon-credit assessments do not include the utilization of pyrolysis co-products such as bio-oil and pyrolysis gas. Incorporating these products as additional energy or material outputs would further reduce net GWP, meaning the mitigation benefits reported here are conservative estimates. Overall, converting unused agro-residues into biochar for cofiring in existing thermal power plants provides a practical, environmentally favorable alternative to uncontrolled open-field burning.

## Conclusions

This study demonstrated that converting agricultural residues into biochar offers a substantially cleaner and more energy-efficient alternative to open-field burning in Korea. Biochar combustion showed higher mass-based emission factors due to carbon and nitrogen concentration during pyrolysis, yet the total annual pollutant emissions decreased markedly because far less mass is required to deliver the same energy output. As a result, system-level emissions of major air pollutants and greenhouse gases were significantly reduced, confirming the environmental advantages of biochar utilization.

From an energy perspective, all examined feedstocks exhibited Energy Return on Investment values above the commonly accepted viability threshold, indicating that biochar production is energetically and economically feasible. Among the residues, apple prunings showed the greatest energy potential and could meaningfully contribute to renewable power generation when co-fired in existing thermal power plants.

These findings suggest that biochar conversion can simultaneously mitigate air pollution, reduce greenhouse gas emissions, and enhance renewable energy production, offering a practical strategy for managing unused agricultural residues in Korea. Because the present assessment excluded potential benefits from using pyrolysis co-products such as bio-oil and syngas, the reported mitigation and economic gains are conservative estimates.

Future research should expand the analysis to include full life-cycle environmental impacts, optimize pyrolysis conditions for diverse agricultural residues, and assess large-scale co-firing scenarios under realistic plant operating conditions. Incorporating spatial data on residue availability and logistics would further strengthen the robustness of national-scale implementation strategies and policy development. In addition, future studies should explicitly incorporate collection and transport processes within an extended system boundary to evaluate their quantitative contribution to overall emissions. Although these processes were excluded in the present study to maintain a focused combustion-stage comparison, integrating logistics-related emissions would provide a more comprehensive life-cycle perspective and support more refined regional deployment strategies.
